# A Proposed Dedicated Breast PET Lexicon: Standardization of Description and Reporting of Radiotracer Uptake in the Breast

**DOI:** 10.3390/diagnostics11071267

**Published:** 2021-07-15

**Authors:** Kanae K. Miyake, Masako Kataoka, Takayoshi Ishimori, Yoshiaki Matsumoto, Masae Torii, Masahiro Takada, Yoko Satoh, Kazunori Kubota, Hiroko Satake, Masahiro Yakami, Hiroyoshi Isoda, Debra M. Ikeda, Masakazu Toi, Yuji Nakamoto

**Affiliations:** 1 Department of Advanced Medical Imaging Research, Graduate School of Medicine Kyoto University, 54 Shogoin Kawahara-cho, Sakyo-ku, Kyoto-City 606-8507, Kyoto, Japan; 2 Department of Diagnostic Imaging and Nuclear Medicine, Graduate School of Medicine Kyoto University, 54 Shogoin Kawahara-cho, Sakyo-ku, Kyoto-City 606-8507, Kyoto, Japan; makok@kuhp.kyoto-u.ac.jp (M.K.); ishimori@kuhp.kyoto-u.ac.jp (T.I.); ynakamo1@kuhp.kyoto-u.ac.jp (Y.N.); 3 Department of Breast Surgery, Graduate School of Medicine Kyoto University, 54 Shogoin Kawahara-cho, Sakyo-ku, Kyoto-City 606-8507, Kyoto, Japan; yoshiaki@kuhp.kyoto-u.ac.jp (Y.M.); masahiro@kuhp.kyoto-u.ac.jp (M.T.); toi@kuhp.kyoto-u.ac.jp (M.T.); 4 Preemptive Medicine and Lifestyle Related Disease Research Center, Kyoto University Hospital, 53 Shogoin Kawahara-cho, Sakyo-ku, Kyoto-City 606-8507, Kyoto, Japan; yakami@kuhp.kyoto-u.ac.jp (M.Y.); sayuki@kuhp.kyoto-u.ac.jp (H.I.); 5 Department of Breast Surgery, Japanese Red Cross Wakayama Medical Center, 4-20 Komatsubara-dori, Wakayama-City 640-8558, Wakayama, Japan; masaet@kuhp.kyoto-u.ac.jp; 6 Yamanashi PET Imaging Clinic, 3046-2 Shimokato, Chuo-City 409-3821, Yamanashi, Japan; ysatoh@ypic.jp; 7 Department of Radiology, Dokkyo Medical University Saitama Medical Center, 2-1-50 Minamikoshigaya, Koshigaya-City 343-8555, Saitama, Japan; kubotard@dokkyomed.ac.jp; 8 Department of Radiology, Nagoya University Graduate School of Medicine, 65 Tsurumai-cho, Showa-ku, Nagoya-City 466-8550, Aichi, Japan; shiroko@med.nagoya-u.ac.jp; 9 Department of Radiology, Stanford University School of Medicine, Breast Imaging, 875 Blake Wilbur Drive, Stanford, CA 94305-5826, USA; dikeda@stanford.edu

**Keywords:** breast cancer, positron emission tomography, dedicated breast PET, breast PET, high-resolution PET, lexicon, ^18^F-fluorodeoxyglucose

## Abstract

Dedicated breast positron emission tomography (dbPET) is a new diagnostic imaging modality recently used in clinical practice for the detection of breast cancer and the assessment of tumor biology. dbPET has higher spatial resolution than that of conventional whole body PET systems, allowing recognition of detailed morphological attributes of radiotracer accumulation within the breast. ^18^F-fluorodeoxyglucose (^18^F-FDG) accumulation in the breast may be due to benign or malignant entities, and recent studies suggest that morphology characterization of ^18^F-FDG uptake could aid in estimating the probability of malignancy. However, across the world, there are many descriptors of breast ^18^F-FDG uptake, limiting comparisons between studies. In this article, we propose a lexicon for breast radiotracer uptake to standardize description and reporting of image findings on dbPET, consisting of terms for image quality, radiotracer fibroglandular uptake, breast lesion uptake.

## 1. Introduction

High-resolution positron emission tomography dedicated for the breast is a modern diagnostic imaging modality used in clinical practice for the detection of breast cancer and assessment of tumor biology, and is expected as a promising tool to support the evolution of molecular imaging [1]. High-resolution breast PET systems are PET systems that examine both breasts using detectors proximal to the breast, which were designed to have a higher spatial resolution and higher sensitivity for emitted radiation compared with conventional whole-body PET systems (WB-PET) [2]. High-resolution PET systems for the breast are generalized into two types: (1) “positron emission mammography (PEM)”, a dual-head system compressing the breast mildly and providing limited-angle tomographic images [3], and (2) a fully tomographic type, representatively ring-type dedicated breast PET (dbPET), a newer generation of breast PET systems acquiring complete three-dimensional data and providing fully tomographic images in any direction [4,5]. While WB-PET has a limited value in evaluating, characterizing, and staging lesions within the breast due to its low spatial resolution, studies have shown that PEM or ring-type dbPET has a superior performance to WB-PET in the detection of breast cancers [6,7,8,9,10]. However, because of its high spatial resolution, dbPET detects and displays radiotracer accumulations that were previously unnoticeable, leading to the detection of a larger number of benign lesions and possibly leading to an increase in false-positive examinations.

Breast cancer is a heterogenous disease entity and has a wide variety of phenotypes. The imaging appearance of breast cancers is diverse with significant overlaps between benign and malignancy. This problem of imaging look-alikes was recognized on conventional X-ray, ultrasound, and dynamic contrast-enhanced magnetic resonance imaging (MRI) studies of the breast, with many publications identifying specific imaging features of breast cancers and useful findings to differentiate or characterize breast lesions, with several investigators generating methods to stratify the probability of malignancy according to the image-based interpretation. Because of the wide variety of naming methods and because of the need to standardize reporting, the American College of Radiology (ACR) organized a group of breast imaging experts to glean known scientific imaging publications for the best-known imaging features of breast lesions based on data. The result was the Breast Imaging-Reporting and Data System (BI-RADS) for mammography, ultrasound, and MRI of the breast. BI-RADS provides terminology, called a *lexicon*, to describe and report imaging findings in a standardized way, and to provide final assessment classifications linked with specific management recommendations [11]. BI-RADS serves as a common language that clearly and consistently conveys findings and assessments from radiologists to referring physicians and patients. Standardized breast background and lesion reporting supports data collection and allows comparison between studies around the world. We believe this concept may be applied to high-resolution PET dedicated for the breast, which allows identifying qualitative features of breast uptake because of its improved spatial resolution. Adding morphological assessment may help differentiate malignant uptake and benign uptake to reduce false positives caused by improved uptake detection in benign breast tissue and/or breast lesions. In 2011, Narayanan et al. first reported the value of feature analysis of breast uptake using PEM with ^18^F-fluorodeoxyglucose (^18^F-FDG), and demonstrated morphological uptake features of additional lesions in patients with breast cancer were significant predictors of malignancy [12]. In recent years, there have been several reports that suggest the usefulness of breast lesion morphological assessment using a ring-type dbPET with ^18^F-FDG [13,14,15,16]. However, to establish and facilitate a comprehensive approach of breast cancer diagnosis using dbPET, there is now a critical need to standardize reporting and descriptors of uptake features.

This paper proposes a dbPET lexicon to describe breast radiotracer uptake on dbPET, and to help in reporting dbPET findings. Many of the terms are similar to the ACR BI-RADS descriptors in order to harmonize with their terminology; however, some descriptors are unique to dbPET given PET’s specific imaging parameters. Typical images of ring-type dbPET with ^18^F-FDG for each lexicon are provided.

## 2. Methods

### 2.1. dbPET Imaging Protocols and Display

#### 2.1.1. Study Protocols and Parameters

Requirements before examinations should follow the general recommendations of PET studies, e.g., at least 4 h fasting for ^18^F-FDG [17,18].

The report should include the type of dbPET device, the imaging acquisition and reconstruction protocols, including the type and injection dose of radiotracer, blood sugar level at injection for ^18^F-FDG, waiting time, emission scan duration, and the reconstruction parameters. Any suboptimal conditions, such as poor positioning and body movement, should be noted in the report, since they potentially influence the degree and the appearance of uptake.

The report should include the study indication, pertinent demographic data, and patient clinical history (examples: gender, patient age, menstrual and menopausal status, medication/exogenous hormone or anti-hormone therapy, breast cancer history, breast intervention and breast reconstruction, and family history of breast cancers and ovarian cancers), as well as any prior examinations and study dates.

#### 2.1.2. dbPET Image Display

Maximum intensity projection (MIP) images of dbPET should be initially assessed with a fixed window width; the recommended lower and upper thresholds are 0 and 3–4, respectively, in standard uptake value (SUV). With implementation of attenuation and scatter corrections, SUV is measurable with some fully tomographic dbPET systems [2,4,5]. It is recommended to display the paired mediolateral (ML) MIP views and the paired craniocaudal (CC) MIP views to compare right and left breasts and add an SUV scale on the side of the images. If the uptake of fibroglandular tissue or breast lesions is intense, it may be necessary to compose additional MIP images with a higher upper threshold to appreciate the dynamic range. Subsequently, tomographic images of each breast should be assessed, adjusting the window to the level to visualize internal properties of the breast or of lesions if needed.

### 2.2. dbPET Lexicon, Version 1.0

The lexicon includes descriptors for image quality, background fibroglandular uptake (bFGU), and breast lesions (Table A1 in Appendix A). Image quality and bFGU are routinely assessed regardless of the presence or absence of breast lesions.

#### 2.2.1. Image Quality

Assessment of image quality is the first step to describe dbPET findings. Noise and field of views (FOV) are included in this assessment.

**Figure 1:**
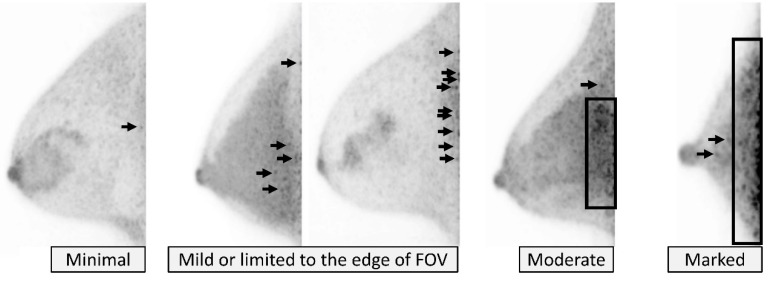
**Image quality: Noise.** From left to right, *minimal*, mild (categorized as “*mild or limited to the edge of field of view (FOV)*”), limited to the edge of FOV (categorized as “*mild or limited to the edge of FOV*”), *moderate*, and *marked* on MIP images. Arrows and rectangles indicate noise.

Noise tends to be relatively higher and the signal-to-noise ratio tends to be lower at the edge of the detector where the gamma-ray coincidences from the annihilation of positrons decrease. Noise level is categorized using the 4-point scale; *minimal*, *mild or limited to the edge of FOV*, *moderate*, and *marked* (Figure 1). *Minimal* noise means absence of noise or almost no noise. *Mild or limited to the edge of FOV* noise includes mild noise, and noise that is relatively strong but localized to the near edge of FOV and does not affect most of the breast. *Moderate* noise is moderately significant noise that extends over the breast parenchyma, not limited to the edge of FOV. *Marked* noise is significant noise that has high potential to mask lesion uptake.

**Figure 2:**
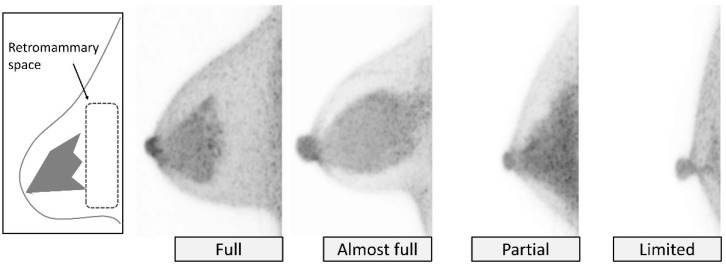
**Image quality: Field of view (FOV).** FOV is classified using retromammary space (see schematics) as hallmark. From left to right, *full*, *almost full*, *partial,* and *limited* on MIP images.

FOV is determined by how much of the breast is included in the FOV of dbPET. The retromammary space is used as a marker to estimate the FOV visualization of breast tissue because the pectoral muscles are usually not included within the FOV of dbPET, which has a wider blind area than mammography. The FOV of dbPET is categorized as *full*, *almost full*, *partial,* and *limited* (Figure 2). Inclusion of the retromammary space is used to categorize a *full*, *almost full*, or *partial* FOV, describing when the retromammary space is visualized entirely (*full*), mostly (*almost full*), or partially to poorly (*partial*). A *limited* FOV means that the breast within the FOV is extremely small due to small breast size, poor positioning, or partial mastectomy, etc. It should be noted that there is a possibility that a part of the breast is still excluded from the FOV even in the case of a *full* FOV.

If there are special notes regarding imaging conditions that can degrade image quality, such as poor positioning, body movement, and change of protocols, we recommend that these be described within the study comments.

#### 2.2.2. Background Fibroglandular Uptake (bFGU)

Fibroglandular tissue usually has higher ^18^F-FDG accumulation compared with fat, thus is seen as FDG-avid structure on dbPET. Background breast fibroglandular uptake (bFGU) is assessed in terms of fibroglandular amount, uptake intensity, distribution, and symmetry. It is important to report bFGU because intense bFGU may hide breast cancers. 

**Figure 3:**
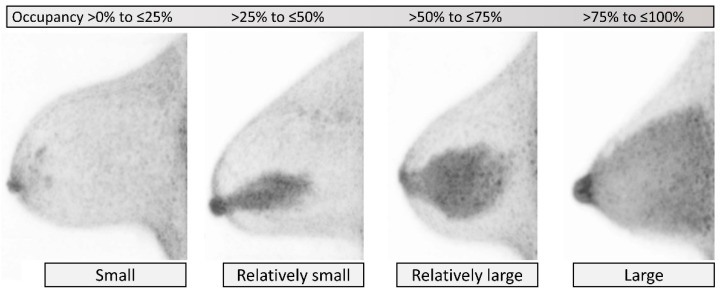
**Background fibroglandular uptake (bFGU): Extent.** From left to right, *small*, *relatively small*, *relatively large*, and *large* on MIP images. No uptake (*none*), another category, is not shown. Amount is classified based on the approximate occupancy of bFGU in the assumed fibroglandular tissue in the breast by volume.

bFGU extent, as a report of the amount of radiotracer-avid background breast tissue in the breast by volume, is categorized with the 5-point scale as *none*, *small*, *relatively small*, *relatively large*, and *large*, corresponding to 0% (*none*), >0% to ≤25% (*small*), >25% to ≤50% (*relatively small*), >50% to ≤75% (*relatively large*), and >75% to ≤100% (*large*), respectively, of the assumed fibroglandular tissue occupying the glandular areas of the breast (Figure 3). No bFGU (*none*) may be rare but can be occasionally seen, e.g., in aged breasts entirely replaced with fat, or in women with mastectomies. When the category *none* is chosen, evaluation of intensity and homogeneity of bFGU will be omitted, and the reason why the breast tissue is absent should be reported.

**Figure 4:**
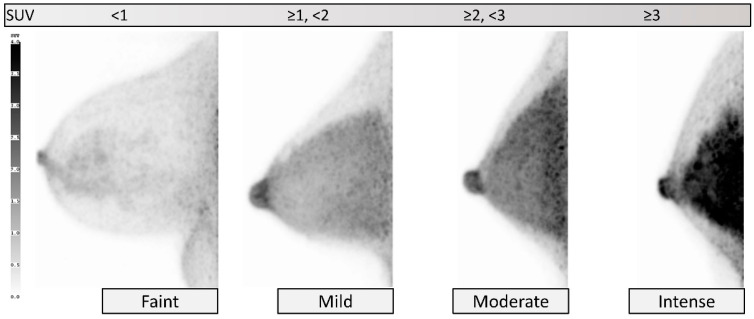
**Background fibroglandular uptake (bFGU): Intensity.** From left to right, *faint*, *mild*, *moderate,* and *intense* on MIP images. Intensity is classified using SUV scale as a guide.

bFGU intensity is categorized as *faint*, *mild*, *moderate*, and *intense*, corresponding to approximately <1, ≥1 and <2, ≥2 and <3, and ≥3 of SUV as a guide, respectively (Figure 4). In heterogeneous bFGU, we suggest that the bFGU be evaluated and reported at the approximate level of areas with the most intense accumulation in the breast. In most cases, bFGU intensity may be classified as *mild*, or less commonly as *moderate* or *faint*. *Intense* bFGU may be seen in lactating breasts or pathological breasts. *Intense* bFGU can mask lesion uptake. If there is significant noise on the image, intensity of bFGU should be assessed in areas that are not affected by noise.

**Figure 5:**
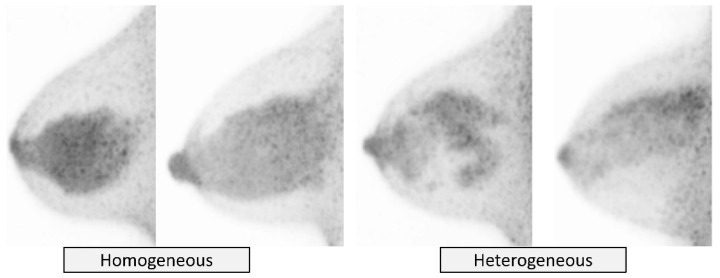
**Background fibroglandular uptake (bFGU): Distribution.***Homogeneous* (**left two**) and *heterogeneous* (**right two**) on MIP images.

Distribution of bFGU in the breast is categorized as *homogeneous* and *heterogeneous* (Figure 5). *Homogeneous* means almost uniform and confluent uptake of the radiotracer-avid fibroglandular tissue. *Heterogeneous* primarily means uneven distribution of radiotracer-avid fibroglandular tissue due to the mixture of fat (i.e., heterogeneous, scattered, island-like, or patchy distribution), but sometimes could be due to uneven uptake level of the fibroglandular tissue itself.

**Figure 6:**
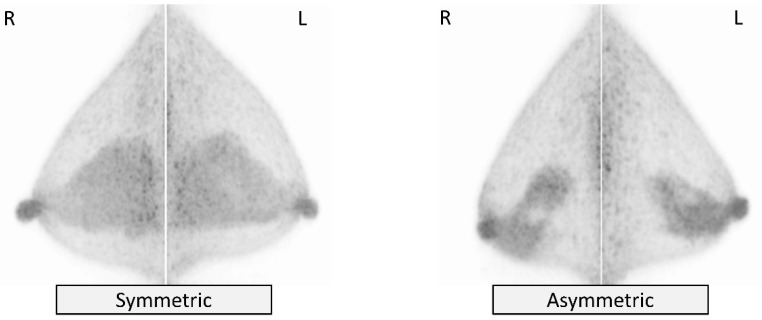
Background fibroglandular uptake (bFGU): Symmetry. *Symmetric* (**left**) and *asymmetric* (**right**) on MIP images.

Symmetry of bFGU in comparison of the left and right breasts is categorized as *symmetric* or *asymmetric* (Figure 6). The routine evaluation of distribution and symmetry may be helpful to allow earlier recognition of a lesion that exhibits unremarkable uptake, which is often difficult to distinguish from bFGU, such as ductal carcinoma in situ (DCIS).

If there are special notes such as post-mastectomy, post-reconstruction, we recommend that these be described in the indication and in the comments.

#### 2.2.3. Breast Lesion

A lesion is defined as an area of abnormal uptake that is unique and different from the bFGU.

Lesions are classified as a *focus*, *mass uptake (MU)*, and *non-mass uptake (NMU)* based on the three-dimensional morphologic features. 

A *focus* is a dot-like small uptake that is difficult to characterize further. Although it is difficult to measure the exact size on PET and size threshold is not strictly defined as a criterion, a *focus* is roughly smaller than 5 mm as a guide in a fixed window of 0 to 3–4 on ^18^F-FDG dbPET.

*Mass uptake (MU)* is uptake larger than 5 mm composed of a three-dimensional uptake finding, which usually has relatively abrupt margins.

*Non-mass uptake (NMU)* is uptake that has a pattern different from that of the bFGU and has a shape that cannot be called a focus or a mass. *NMU* includes uptake with its boundary intensity shifting gradually toward the intensity of the background or is composed of tightly gathered multiple foci in one area.

For reporting of a breast lesion, we propose describing the lesion location and uptake intensity using the following descriptors. 

**Figure 7:**
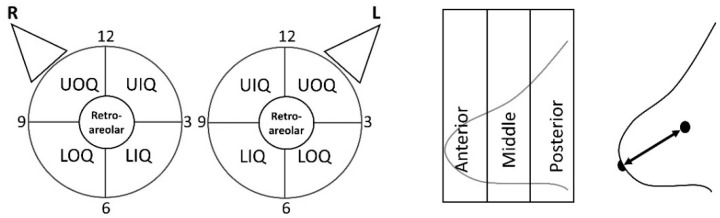
**Lesion: Location.** Schematics of *quadrant* and *clock-face location* (**left**), *depth* (**middle**), and *the distance of the lesion from the nipple* (**right**). *UIQ* = upper inner quadrant, *LIQ* = lower inner quadrant, *UOQ* = upper outer quadrant, *LOQ* = lower outer quadrant.

Descriptions of the lesion location include *breast laterality* (right or left breast), *breast quadrant* (upper outer quadrant towards the axilla (*UOQ*), upper inner quadrant (*UIQ*), lower outer quadrant (*LOQ*), lower inner quadrant (*LIQ*), and around or posterior to the nipple (*retroareolar*)), *clock-face location, lesion depth* (anterior, middle, and posterior third of the breast), and *the distance of the lesion from the nipple* (Figure 7).

Specifically, the lesion *clock-face location* is described as if the patient is facing the interpreter and each breast is on the face of a clock. In both the right and left breasts, lesions at the cranial aspect of the breast at the level of the nipple would be described as at the 12 o’clock position, while lesions at the caudal aspect of the breast at the level of the nipple would be described as at the 6 o’clock position of the breast. However, lesions in the right breast in the upper outer quadrant towards the axilla (UOQ) would be at the 10 o’clock position, whereas lesions in the UOQ of the left breast would at the 2 o’clock position.

**Figure 8:**
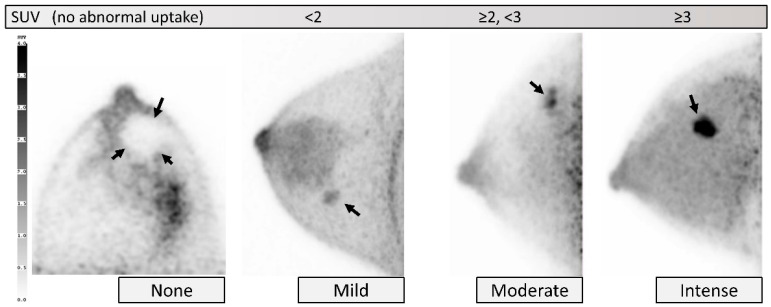
**Lesion: Intensity.** From left to right, *none* (cyst; tomographic image), *mild* (category 2 on ultrasound; MIP), *moderate* (intraductal papilloma; MIP), and *intense* (invasive ductal carcinoma; MIP). Intensity is classified using SUV scale as a guide. Arrows indicate target lesions.

Qualitative uptake intensity level is determined based on the visual assessment of the intense parts of the lesion using SUV scale as a guide, and categorized as no abnormal uptake or *none*, *mild* (approximately SUV < 2), *moderate* (approximately SUV ≥ 2 and <3), and *intense* (approximately SUV ≥ 3) (Figure 8). Quantitative assessment, using SUV, can be applied but is not mandatory. It should be noted that SUVmax is a pixel value, which can overestimate uptake degree and be enhanced by the effect of noise, especially on high-resolution dbPET images. *None* or no abnormal intensity in the location of a known breast lesion may be difficult to be identified using dbPET alone, and should be reserved to assess a specific lesion in comparison with other imaging modalities. As an example, if there are breast calcifications highly suspicious for malignancy on a mammogram and the dbPET shows no abnormal uptake in the same location, one may report the intensity of the dbPET in the location of the known suspicious finding as having “no abnormal uptake” or that the uptake intensity of the dbPET is “*none*” in the location of the known suspicious finding.

As described earlier, all lesions should be initially assessed on MIP using a fixed window (recommendation: 0 to 3–4 for ^18^F-FDG), and then further evaluated with tomographic images using an adjusted dynamic range to see detailed ^18^F-FDG uptake pattern within tumors. Particularly, for malignant lesions with intense uptake, it is important to adjust the dynamic range to assess the lesion morphology and internal uptake details.

##### Focus

*Focus* is a dot-like area of uptake that is difficult to characterize further, and is usually smaller than 5 mm in a fixed window of 0 to 3–4 for ^18^F-FDG. A focus can include noise, unless noise is reliably differentiated from true abnormal uptake.

**Figure 9:**
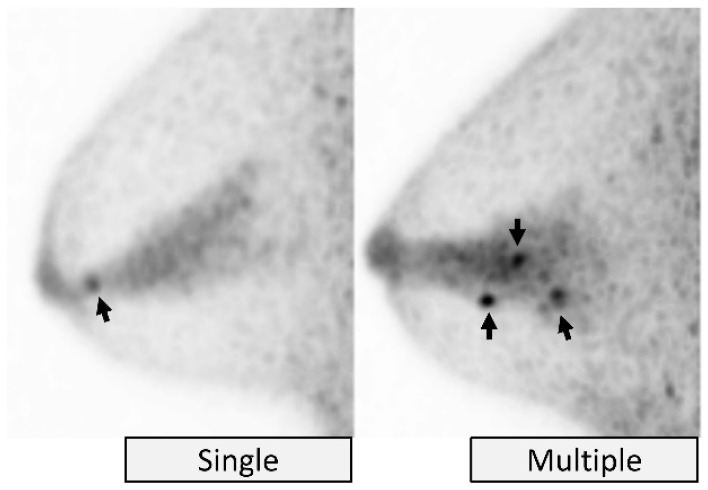
**Focus.***Single* (**left**, intraductal papilloma), and *multiple* (**right**, usual ductal hyperplasia) on MIP images. Arrows indicate foci.

The focus descriptor is subdivided into a *single focus* or *multiple foci* (Figure 9). A *single focus* is one, solitary dot-like area of uptake. Two foci are described separately as a different single focus for each. The term “*multiple foci*” describes three or more foci scattered in the breast independently from the distribution of glandular tissue lobes. If three or more foci gather closely together or appear along a glandular lobe, they are described as NMU instead of multiple foci.

##### Mass Uptake (MU)

*Mass uptake* (*MU*) describes a three-dimensional mass-like area of uptake that usually has distinct edges compared to the bFGU, and is described by its shape and internal pattern.

**Figure 10:**
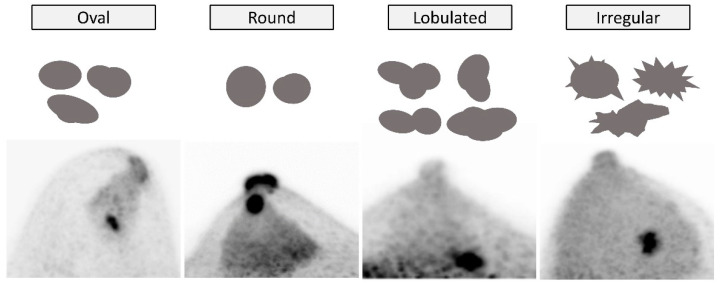
**Mass uptake: Shape.** From left to right, *oval* (no malignancy on biopsy), *round* (no histological diagnosis), *lobulated* (no histological diagnosis), and *irregular* (invasive ductal carcinoma) on MIP images with schematics.

A mass shape is categorized as *oval*, *round*, *lobulated*, or *irregular*, based on the features of both the overall mass uptake shape and margins (Figure 10). *Oval* or *round* mass uptake has smooth margins, and may be accompanied with up to two mild undulations with a dull angle. *Lobulated* mass uptake also has smooth margins but has apparent lobulations with clear sharp angles with three or more undulations. *Irregular* masses have irregular margins (sawtooth, spiculated, angular, or indistinct) or are those that have irregular extensions into the surrounding tissue.

**Figure 11:**
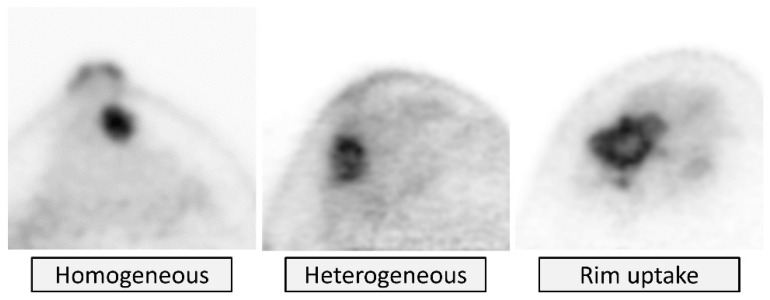
**Mass uptake: Internal pattern.** From left to right, *homogeneous* (no histological diagnosis), *heterogeneous* (invasive ductal carcinoma), and *rim uptake* (invasive ductal carcinoma) on tomographic images.

The mass internal pattern is categorized as *homogeneous*, *heterogeneous*, or *rim uptake* (Figure 11). *Homogeneous* uptake describes uptake that is of uniform intensity throughout the lesion. *Heterogeneous* uptake describes variable uptake intensity within the lesion. *Rim uptake* describes unique heterogeneous moderate or intense uptake along the edges of the lesion or along the lesion “rim” with the central area of the lesion showing no or mild abnormal uptake. *Rim uptake* includes either a full rim (uptake highest along all lesion edges) or a partial rim (higher uptake along only part of the lesion edge). Heterogeneous uptake within and up to the lesion edge other than rim uptake is categorized as *heterogeneous*.

##### Non-Mass Uptake (NMU)

*Non-mass uptake (NMU)* is defined as abnormal uptake unique and different from that of the background fibroglandular glandular tissue (bFGU), larger than 5 mm and with a shape that cannot be called a mass or a focus. NMU includes uptake with an indistinct boundary and an accumulation intensity shifting gradually lower toward the bFGU (exhibiting one or more uptake grades lower than that of central part). NMU also describes multiple foci that are closely gathered together. NMU descriptors include both the NMU distribution and internal accumulation pattern.

**Figure 12:**
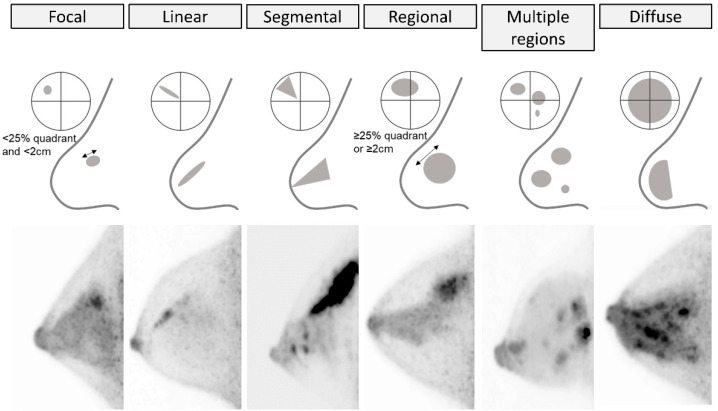
**Non-mass uptake (NMU): Distribution.** From left to right, *focal* (fibrocystic disease), *linear* (invasive ductal carcinoma), *segmental* (ductal carcinoma in situ and invasive ductal carcinoma), *regional* (radial sclerosing lesion), *multiple regions* (invasive ductal carcinoma), and *diffuse* (lymphocytic lobulitis) on MIP images with schematics. The double-headed arrows indicate the diameter of a lesion.

**Figure 13:**
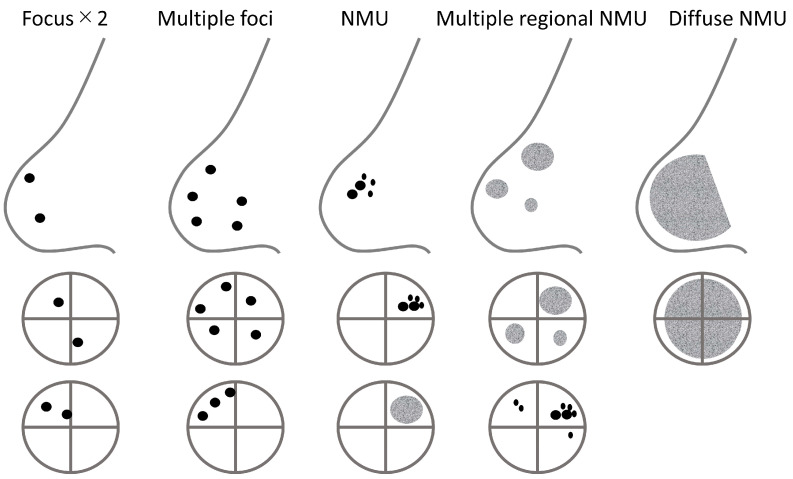
Schematics of focus, multiple foci, NMU, multiple regional NMU, and diffuse NMU.

NMU distribution descriptors include *focal*, *linear*, *segmental*, *regional*, *multiple regions*, and *diffuse* (Figure 12). *Linear* or *segmental* distribution may indicate extension of lesions along breast glandular lobes and ducts. *Focal* or *regional* distribution means localized NMU; *focal* = less than 25% of quadrant, *regional* = equal or more than 25% of quadrant. *Multiple regional* NMU distribution is defined as NMU that scatters in three or more locations, independently from the distribution of glandular lobes. When using this description for uptake consisting of mixtures of foci, the uptake pattern should include at least one location of NMU. *Diffuse* NMU describes NMU that almost continuously and widely extends over the whole breast. Examples of focus, multiple foci, NMU, multiple regional NMU, and diffuse NMU are illustrated in Figure 13.

**Figure 14:**
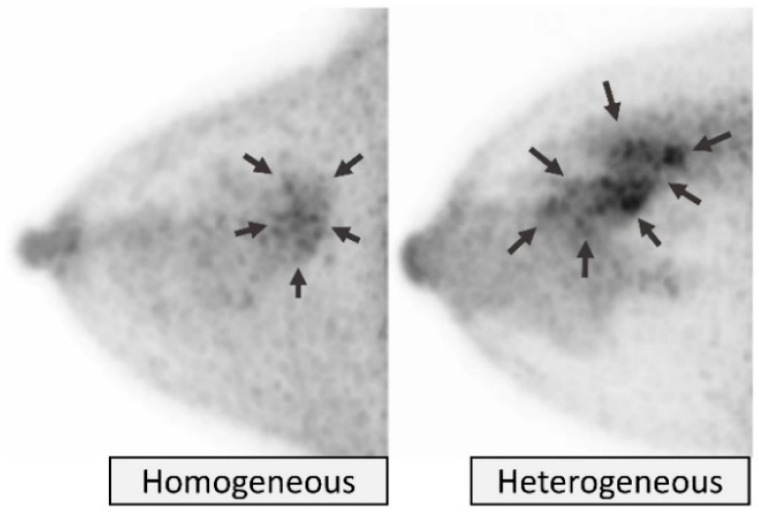
**Non-mass uptake (NMU): Internal pattern**. *Homogeneous* (**left**, ductal carcinoma in situ) and *heterogeneous* (**right**, no malignancy on biopsy). Arrows indicate NMU.

The internal pattern of NMU is categorized as *homogeneous*, characterized by uniform uptake, or *heterogeneous*, characterized as non-uniform uptake (Figure 14).

##### Associated Findings

Features associated with lesion uptake, when visualized, include nipple retraction, skin retraction, nipple uptake, skin uptake, and pectoral muscle/chest wall uptake.

##### Special Findings

*Cyst-like uptake* describes a clear circular or ball-like uptake defect with or without surrounding uptake. This category is selected when a simple cyst, complicated cyst, or fat necrosis is strongly suspected or known by other imaging methods. If the uptake defect is not sharply marginated and difficult to define, or has rim uptake in a location without a known cyst, the uptake should be categorized as a mass with rim uptake.

A *breast implant* describes accumulation defects due to known breast implants. If there is elevated uptake around the implant, the increased uptake should be considered abnormal and described in the section of breast lesions.

## 3. Discussion

The clinical usefulness of morphological features of breast uptake on ring-type dbPET has been demonstrated by several reports. Satoh et al. found that mass uptake was significantly associated with malignancy compared to focus in 40 unexpected uptake lesions in 35 patients [13]. Sasada et al. showed that mass and focal/segmental non-mass lesions were significantly associated with breast cancer among 910 abnormal findings in 709 patients with breast cancer [14]. Masumoto et al. reported that heterogeneity in tumor uptake was associated with higher nuclear grade and higher Ki67 in breast cancers [15]. Sakaguchi et al. showed that rim uptake was associated with higher Ki67 and triple-negative tumor status [16]. Although the definitions of uptake features in these studies were not uniform, these reports suggest the addition of morphological feature analysis may be helpful in characterizing breast uptake, and could be a clue to resolve issues of false-positive reports caused by uptake in benign breast conditions. By collecting data using this lexicon with unified reporting criteria, we believe that features of breast cancer uptake may be clarified and the evidence of predictive values of each uptake feature may be established.

The ACR BI-RADS Atlas recommends that the interpretation of imaging findings use a final assessment category consisting of categories 0 to 6, which represents the probability of malignancy and is linked to recommendations of management [11]. We encourage the adoption of this same system in the future, once the reproducibility and the usefulness of this lexicon has been validated and established. 

It is an urgent task to identify the probabilities of malignancy of dbPET findings and establish the interpretational criteria of dbPET in breast cancer screening, which may be possible with the use of the standardized lexicon. Mammography and ultrasonography are conventional imaging modalities with established roles in the early diagnosis of breast cancer; however, some diagnostic limitations are known, such as mammography being less sensitive in dense breasts and ultrasonography being operator-dependent. MRI is known as the most sensitive modality for breast cancer, but has frequently resulted in false-positive diagnoses. Previously Berg et al. demonstrated that PEM with ^18^F-FDG was much more sensitive than conventional imaging (mammography and often targeted ultrasonography) (41% vs. 21% at lesion level) in the detection of additional cancers in the ipsilateral breast in 388 women with newly diagnosed breast cancer [19]. They also showed that PEM had improved specificity (79.9% vs. 65.6%) compared with MRI, and was less likely to prompt unnecessary biopsies. A combination of high-resolution PET systems with standard breast imaging modalities may improve the breast cancer detection. In addition, with the development and application of various PET tracers, high-resolution PET systems will further extend the roles of imaging in breast cancer diagnosis.

In our dbPET lexicon, uptake intensity levels are graded using SUV scale as a guide. This grading system is designed to distinguish between relatively low levels of uptake, in order to differentiate the uptake levels of the normal fibroglandular tissue, that is, bFGU, as well as to identify and stratify breast lesions with mild or moderate uptake, especially those that need to be differentiated from small or low-FDG-avid breast cancers, on ^18^F-FDG dbPET. According to a study by Nishimatsu et al., SUVmax of the contralateral breasts considered as normal in 140 patients with breast cancer ranged from 0.7 to 3.7 with a mean value of 1.8 on ^18^F-FDG dbPET [20], supporting that our grading system for bFGU intensity is set to reasonable SUV levels. They also reported that SUVmax of 179 breast cancers widely varied with a range of 0.9 to 67.3. Sueoka et al. showed that the luminal A-like subtype, smaller size, and lower grade were significantly associated with lower SUVmax on ^18^F-FDG dbPET [10]. Our lexicon is expected to contribute to picking up lesions with subtle contrast, including small or low-FDG-avid breast cancers, and further differentiating of these lesions.

The dbPET lexicon was created primarily assuming the use of ring-type dbPET with ^18^F-FDG. Previously, a lexicon for PEM with ^18^F-FDG [12] and for ^99m^Tc-sestamibi dual-head gamma breast imaging [21] was proposed. Since ring-type dbPET has an advantage over PEM in that it provides complete three-dimensional images and tomographic images in any direction while quantifying SUVs, our descriptors include specific and detailed terms representing high-resolution three-dimensional breast lesion morphology and accumulation intensity using SUV as a guide. The concept and the information obtained by high-resolution PET systems, however, have much in common, and most parts of our lexicon may be potentially applicable to the other high-resolution PET devices, including PEM, as well as to various tracers. More research validating this reporting system’s reproducibility and applicability is needed.

## 4. Conclusions

In this article, we propose a reporting lexicon of dbPET (version 1.0.) to standardize the reporting of dbPET and dbPET-detected breast findings, with an emphasis on descriptors of morphological features of normal breast fibroglandular tissue and breast lesions. With the advent of PET technology advances, the comprehensive assessment of breast uptake is possible based on three-dimensional morphology feature and uptake assessments.

## Appendix A

**Table A1:** dbPET lexicon, version 1.0.

**Image quality**			
Noise		a. Minimal	Absence of noise or almost no noise
		b. Mild or limited to the edge of FOV	Mild noise or noise localized only to the near FOV edge
		c. Moderate	Moderate noise
		d. Marked	Significant noise, which can mask lesions
FOV		a. Full	The whole radiotracer-avid fibroglandular gland is completely contained within the FOV. The entire retromammary space is identified.
		b. Almost full	Most of the radiotracer-avid fibroglandular gland is included. The retromammary space is partially interrupted.
		c. Partial	The radiotracer-avid fibroglandular gland is partly out of FOV. The retromammary space is partly or poorly visualized.
		d. Limited	The breast in the FOV is visualized very limitedly.
Notes		If there are special notes (poor positioning, body movement, artifacts, and change of protocols (radiotracer dose, waiting time, emission scan duration, reconstruction parameters, etc.)), describe.	
**Background fibroglandular uptake (bFGU)**			
Extent		0. None	No radiotracer-avid fibroglandular tissue. When this category is chosen, subsequent evaluation of intensity and homogeneity of FGU will be skipped.
		a. Small	A small amount of radiotracer-avid fibroglandular tissue, occupying approximately >0% to ≤25% of the assumed fibroglandular tissue in the breast by volume
		b. Relatively small	A relatively small amount of radiotracer-avid fibroglandular tissue, occupying approximately >25% to ≤50% of the assumed fibroglandular tissue in the breast by volume
		c. Relatively large	A relatively large amount of radiotracer-avid fibroglandular tissue, occupying approximately >50% to ≤75% of the assumed fibroglandular tissue in the breast by volume
		d. Large	A large amount of radiotracer-avid fibroglandular tissue, occupying approximately >75% to ≤100% of the assumed fibroglandular tissue in the breast by volume
Intensity		a. Faint	Very low intensity (Approximately SUV <1 for ^18^F-FDG).
		b. Mild	Low intensity (Approximately SUV ≥1 to <2 for ^18^F-FDG)
		c. Moderate	Mildly increased uptake. (Approximately SUV ≥2 to <3 for ^18^F-FDG)
		d. Intense	Extremely increased uptake (Approximately SUV ≥3 for ^18^F-FDG)
Distribution		a. Homogeneous	Almost uniform and confluent uptake of the radiotracer-avid fibroglandular tissue
		b. Heterogeneous	Heterogeneous fibroglandular tissue uptake, due to the mixture of fat (i.e., heterogenous, scattered, island-like or patchy) or uneven uptake level in the fibroglandular tissue itself.
Symmetry		a. Symmetric	Almost symmetrical pattern between the right and left breasts
		b. Asymmetric	Asymmetrical pattern between the right and left breasts
Notes		If there are special notes such as post-mastectomy, post-reconstruction, describe.	
**Lesion type**			
**Focus;** Dot-like accumulation. Size threshold is not defined as a criterion, but focus is roughly smaller than 5 mm as a guide.			
Single or multiple		a. Single	Single focus
		b. Multiple	Three of more foci, scattered in the breast
**Mass uptake (MU);** Three-dimensionally mass-like uptake			
Shape		a. Oval	Elliptical or egg-shaped uptake (may have up to 2 mild undulations with a dull angle)
		b. Round	Spherical, circular, round uptake (may have up to 2 mild undulations with a dull angle)
		c. Lobulated	Lobulated uptake with clear undulations with sharp angles, or 3 or more undulations
		d. Irregular	Uptake with irregular margins (sawtooth, spiculated, angular, or indistinct) or lesions that develop irregular extension
Internal pattern		a. Homogeneous	Almost homogeneous accumulation
		b. Heterogeneous	Heterogeneous accumulation
		c. Rim uptake	Rim-shaped accumulation with a loss or marked reduction of accumulation in the central part. The rim includes full rim and partial rim.
**Non-mass uptake (NMU);** A pattern different from that of the background fibroglandular tissue, and has a shape that cannot be called a mass or focus			
Distribution		a. Focal	Less than 25% of quadrant or less than 2 cm in diameter
		b. Linear	Linear toward the nipple. It may be tubular or branched.
		c. Segmental	Distribution of triangles with one apex towards the nipple side, suggesting distribution of glandular lobes and ducts
		d. Regional	25% or more of quadrant or 2 cm or more in diameter
		e. Multiple regions	Multiple uptake areas in 3 or more locations, independent from the distribution of glandular lobes. Focus can be included, but uptake pattern at least one location should be NMU.
		f. Diffuse	Diffuse accumulation
Internal pattern		a. Homogeneous	Almost homogeneous
		b. Heterogeneous	Heterogeneous
**Common assessment for lesion**			
Location	Laterality	Right	
		Left	
	Quadrant	UOQ	Upper outer quadrant
		LOQ	Lower outer quadrant
		UIQ	Upper inner quadrant
		LIQ	Lower inner quadrant
		Retroareolar	Around or posterior to the nipple
	Clock face		Clock-face position
	Depth	Anterior	1/3 anterior of the breast within FOV
		Middle	1/3 middle of the breast within FOV
		Posterior	1/3 posterior of the breast within FOV
	Distance from the nipple		Distance of the lesion from the nipple
Qualitative uptake intensity		a. None	No abnormal accumulation
		b. Mild	Low intensity (Approximately SUV < 2 for ^18^F-FDG).
		c. Moderate	Moderate uptake (Approximately SUV ≥ 2, <3 for ^18^F-FDG)
		d. Intense	Increased uptake (Approximately SUV ≥ 3 for ^18^F-FDG)
Quantitative uptake intensity			Qualitative radiotracer uptake values, i.e., SUV
Associated findings		Nipple retraction	
		Skin retraction	
		Nipple uptake	
		Skin uptake	
		Pectoral muscle/chest wall uptake	
**Special findings**			
		Cyst-like uptake	A clear circular uptake defect with or without surrounding uptake. This category is selected when cyst or fat necrosis is strongly suspected or known.
		Breast implant	Accumulation defect due to implant

dbPET, dedicated breast positron emission tomography; FOV, field of view; SUV, standard uptake value; ^18^F-FDG, ^18^F-fluorodeoxyglucose.
